# Supplementary material for: Data-driven prior elicitation for Bayes factors in Cox regression for nine subfields in biomedicine
Source: PLoS One. 2025 May 23;20(5):e0322144. doi: 10.1371/journal.pone.0322144 (PMC12101706; doi:10.1371/journal.pone.0322144)
Supplement: Attachment — Submitted filename: PONE-D-24-18152_Review.pdf [file pone.0322144.s001.pdf]

Summary:

The manuscript proposes „informed default“ prior distributions for the beta coefficient in Cox regression in nine subfields of biomedical research. The priors are based on the analysis of Cox regression coefficients in a large corpus of biomedical studies.

Review:

Finding suitable prior distributions in Bayesian modeling is challenging, and the present manuscript proposes a reasonable way to address this challenge. The methodology for prior specification followed in the manuscript has been applied in similar ways before, but to my knowledge, not for Cox regressions in biomedical science. Given the popularity of survival models and the increasing uptake of Bayesian statistics across scientific fields, I believe that the manuscript can make a valuable contribution to the literature. However, I also have several concerns about the paper, particularly about the validity of the data extraction procedure and the appropriateness of the application example and the simulation study. I will go into more detail on my concerns below and I recommend that the authors address these concerns in a revised version of the manuscript.

(1) I appreciate that the authors used such a large corpus of studies as a basis for prior specification. However, I am concerned that the way the regression coefficients were extracted from the literature may introduce bias into the results. Specifically, estimates were extracted from articles that mentioned the method as well as estimates and confidence intervals in the abstract. This makes me wonder: How often does it happen at all that the specific statistical method is mentioned in the abstract (rather than only in the results section of the article)? To what extent do articles that mention the method (and specific estimates) in the abstract differ systematically from articles that do not mention these details in the abstract? It seems like the extraction method may give preference to articles with structured abstracts (in my view, the most minor bias since the abstract structure is often guided by journal policies rather than by article content), but potentially also to articles where a statistician/methodologist was involved (hence the stronger focus on statistical methods and outcomes), or to articles where a significant result or large effect size was found (something "worth mentioning" in an abstract). It would be good if the authors could run some robustness analyses to check the unbiasedness of extracted effect sizes (e.g., compare full-text search for Cox regression in some journal issues to abstract-only search and compare the results to get a feeling for the potential bias).

(2) Related to my previous point, I think that there are a few details about the extraction method that could be made clearer in the manuscript. Specifically, the authors mention that they extracted articles from "top journals" in the respective fields, but the definition of a "top journal" remained vague. How did the authors ensure that no predatory (pseudo-peer reviewed) journals were part of the selection? Moreover, when explaining the search algorithm using regular expressions, the examples include different versions of "HR". How can we be sure that "HR" always refers to a hazard ratio and isn't an abbreviation of something else (e.g., heart rate) and that the reported numbers hence refer to a different quantity? And finally, the extraction method for the standard error assumes two-sided confidence intervals. Intuitively, it makes sense that this would be the standard, but wouldn't it be possible that some articles report one-sided confidence intervals (e.g., with a one-sided test of treatment effectiveness?).

(3) For me, the application example presented on pp. 9-12 does not fall into the intended application domain of the specified prior distributions. The prior distribution was elicited for the beta coefficient in Cox regressions, but in the presented application example, the prior distribution was applied to the fixed effect in a meta-analysis across five Cox regressions. I

would argue that the specified prior distribution is not a good match for the analysis presented in the application example because the meta-analytic effect size estimate tends to be less extreme than the effect size estimate in a single study. Specifically, we can expect that due to sampling variability, the effect size in a single (potentially small) study is more extreme than the combined estimate across multiple studies (weighed by the study sample size). An appropriate prior specification procedure for meta-analytic effect size estimates in Cox regressions that is similar to the described procedure would therefore, in my view, not specify the prior based on a corpus of individual studies, but on a corpus of meta-analyses. I would therefore highly recommend to exchange the example application in the paper with a Bayesian Cox regression in a single study.

(4) In addition to my previous point, I believe that the presented application example falls short of showcasing the advantages of the methodology and the recommended best practices for using the informed prior distribution. The authors repeatedly mention in the introduction of the manuscript that two advantages of the Bayesian statistical framework are the possibility of quantifying evidence for the null hypothesis and the possibility of flexible sequential designs. I believe that it would be a great opportunity to showcase these advantages in the application example by using a sequential hypothesis test with the specified priors and quantifying the evidence for H0 and H1. Moreover, the discussion section of the manuscript mentions that sensitivity analyses for prior distributions are considered best practice, yet the application example does not include sensitivity analyses. I think that here, too, it would be a good opportunity to showcase this best practice in the application example using the specified prior distributions of different (adjacent?) subfields as well as a default prior distribution.

(5) If my calculations are right, the simulation study presented on p. 11 / Figure 3 is unnecessary. The authors decide to assume the same sample size for all studies. This means that Equation 10 can be drastically simplified and basically yields an analytic result for the simulations. Specifically, the index j is unnecessary to describe the sample size n (n is a constant), since all studies are assumed to have the same sample size. Therefore, the terms under the square root can be simplified to:

$$\sum(n-1)\theta_j^2 = (n-1)\sum\theta_j^2$$
$$\sum n\left(\omega_j - \mu^p\right) = n\sum\left(\omega_j - 0\right)^2 = n\sum\omega_j^2$$
$$\sum_{i=1}^{K} n_j - 1 = nK - 1$$

With this simpler form of the equation, it also becomes relatively clear that the "mirroring" process in the manuscript has no effect on the results. We can simply write the equation in terms of original effect sizes. For each study, the following is true (note that SE(beta) = SE(-beta)):

$$(n-1)\sum\theta_j^2 = (n-1)\left(SE_j^2 + SE_j^2\right) = 2SE_j^2(n-1)$$
$$n\sum\omega_j^2 = n\left(\beta_j^2 + \left(-\beta_j\right)^2\right) = 2n\beta_j^2$$

This means, we can express Equation 10 as:

$$\sqrt{\frac{2(n-1)\sum SE_j^2 + 2n\sum\beta_j^2}{n \cdot 2N - 1}} = \sqrt{\frac{(n-1)\sum SE_j^2 + n\sum\beta_j^2}{nN - 1}}$$

From this, we can see that the influence of the sample size of a single study, n, influences the comparative weighting of SE and beta in the equation: In the most extreme cases, beta gets

weighed twice as much as SE (for n = 2) or beta and SE obtain equal weights (for n=Inf), respectively. We can simply plug in the assumed sample sizes (10:10000), as well as the sum of SEs and sum of betas into the equation to analyze the sensitivity of results.

(6) In my view, a more sensible way to test the sensitivity of results to the sample sizes would be to draw sets of different sample sizes from realistic distributions (I believe that there are papers on sample size / statistical power development across time in the biomedical sciences?) and use these to determine sigma^p. This way, n would actually differ per study and different studies would be weighed differently in each iteration of the simulation. To make it more realistic, the SEs of individual studies should probably also be negatively correlated with the sample size in the simulation (higher sample size, lower SE).

(7) In the discussion section, the authors write that "it is possible that certain journals were systematically underrepresented in our results". To me, this sounds like an empirical question that could be answered using the extracted data? If the proportion of articles selected from each journal within a field does not decrease at the same rate throughout the selection process (e.g. the step indicated in Table 1), then there is some bias. This could for example be represented in some sort of Sankey diagram.

(8) In the introduction, I think it could be made clearer that there is a rich tradition of sequential designs in frequentist statistics, too. In particular, in my knowledge, clinical biomedical studies are one of the key application domains of Group Sequential designs. I agree with the authors that Bayesian methods are more flexible, but I think this point may need a bit of a more nuanced discussion.

(9) Also in the introduction, it could be made clearer that the specification of prior distributions is not only necessary in the context of Bayesian hypothesis testing with Bayes factors, but also in the context of Bayesian parameter estimation. I understand that the focus of the article is on Bayes factors, but to readers with little experience in Bayesian statistics, this might lead to misunderstandings.

(10) Generally, I believe that in practice "informed default" priors are unfortunately often applied outside of their intended application domain (e.g., as has happened to the Oosterwijk prior that is applied outside of social psychology and wasn't even initially intended as a default). Of course, how the priors will be used in practice is outside of the control of the authors. However, I believe that a thorough discussion of the limitations of recommended use in the discussion section of the manuscript could be beneficial to restrict their use to reasonable applications. For example, I would argue that the specified priors are not ideal for meta-analytic effect size parameters (see my point above) and that they should not be used outside of Cox regression (this may be obvious to the authors, but maybe not to non-experts in Bayesian analyses). Moreover, if more field-specific result summaries exist (e.g., Cochrane meta-analysis on breast cancer treatments), and a new analysis is run in this particular field (e.g., new breast cancer treatment), then it might be good to create a more informed prior distribution based on these field-specific results.

(11) Another aspect that I believe warrants further discussion is how the mix of results for the null- and alternative hypothesis in the literature influences the specified prior distributions. Specifically, the specified prior distributions are intended for use under the alternative hypothesis. However, unless there is severe publication bias, it can be assumed that the literature contains effect sizes that are generated under the null- and alternative hypothesis. From a theoretical standpoint, I am wondering: Should effect sizes that were generated under the null hypothesis really be included to specify a prior for the alternative hypothesis?

(12) Related to my previous point, I believe that the topic of how publication bias might have influenced the specified prior distributions warrants further discussion. Ideally, it would be

great to see some p-curves or z-curves together to contextualize the results, but since p-values were not extracted, I understand that this is difficult.

(13) In the results section, it would be great if the authors could include some descriptive statistics of the betas and SEs in the different subfields, and perhaps also the sum of beta^2 and the sum of SE^2 for each discipline. Given the extent to which the results depend on these statistics (see also my earlier points), it would be great to have some more information available in the manuscript.

(14) Reading the manuscript, I was wondering a little what prior distribution the authors would recommend for directional tests. For example, if I assume that a new treatment is better than an old treatment, I would want to test a directional test on the beta parameter. Should I just use the specified distribution for my field and truncate it at zero (e.g., a truncated Normal(0, 0.96) for cardiology)? Or would it be better to use a non-central informed prior distribution? What could be the advantages/disadvantages of each?

Signed

Angelika Stefan
