# Supplementary material for: Data-driven prior elicitation for Bayes factors in Cox regression for nine subfields in biomedicine
Source: PLoS One. 2025 May 23;20(5):e0322144. doi: 10.1371/journal.pone.0322144 (PMC12101706; doi:10.1371/journal.pone.0322144)
Supplement: Attachment — Submitted filename: Response_to_Reviewers_auresp_2.docx [file pone.0322144.s003.docx]

Dear Dr. Haunschild,

Thank you for considering our manuscript “Data-driven prior elicitation for Bayes factors in Cox regression for nine subfields in biomedicine” for publication in PLOS ONE. We have read your and the reviewers’ comments carefully. Below you find our responses. The reviewers’ comments are shown in regular font and our responses are shown in bold font. Our revision contains a version of our manuscript without track changes (i.e., a clean version with all changes already incorporated) and a version of our manuscript with track changes. For the latter, track changes that correspond to additions are shown in blue font and track changes that correspond to removals are shown in red font.

We look forward to your comments.

Kind regards,

Maximilian Linde

Laura Jochim

Jorge N. Tendeiro

Don van Ravenzwaaij

PONE-D-24-18152R1

Data-driven prior elicitation for Bayes factors in Cox regression for nine subfields in biomedicine

PLOS ONE

Dear Dr. Linde,

Thank you for submitting your manuscript to PLOS ONE. After careful consideration, we feel that it has merit but does not fully meet PLOS ONE’s publication criteria as it currently stands. Therefore, we invite you to submit a revised version of the manuscript that addresses the points raised during the review process.

Please submit your revised manuscript by Mar 01 2025 11:59PM. If you will need more time than this to complete your revisions, please reply to this message or contact the journal office at plosone@plos.org. When you're ready to submit your revision, log on to https://www.editorialmanager.com/pone/ and select the 'Submissions Needing Revision' folder to locate your manuscript file.

Please include the following items when submitting your revised manuscript:

A rebuttal letter that responds to each point raised by the academic editor and reviewer(s). You should upload this letter as a separate file labeled 'Response to Reviewers'.

A marked-up copy of your manuscript that highlights changes made to the original version. You should upload this as a separate file labeled 'Revised Manuscript with Track Changes'.

An unmarked version of your revised paper without tracked changes. You should upload this as a separate file labeled 'Manuscript'.

If you would like to make changes to your financial disclosure, please include your updated statement in your cover letter. Guidelines for resubmitting your figure files are available below the reviewer comments at the end of this letter.

If applicable, we recommend that you deposit your laboratory protocols in protocols.io to enhance the reproducibility of your results. Protocols.io assigns your protocol its own identifier (DOI) so that it can be cited independently in the future. For instructions see: https://journals.plos.org/plosone/s/submission-guidelines#loc-laboratory-protocols. Additionally, PLOS ONE offers an option for publishing peer-reviewed Lab Protocol articles, which describe protocols hosted on protocols.io. Read more information on sharing protocols at https://plos.org/protocols?utm_medium=editorial-email&utm_source=authorletters&utm_campaign=protocols.

We look forward to receiving your revised manuscript.

Kind regards,

Robin Haunschild

Academic Editor

PLOS ONE

Journal Requirements:

Please review your reference list to ensure that it is complete and correct. If you have cited papers that have been retracted, please include the rationale for doing so in the manuscript text, or remove these references and replace them with relevant current references. Any changes to the reference list should be mentioned in the rebuttal letter that accompanies your revised manuscript. If you need to cite a retracted article, indicate the article’s retracted status in the References list and also include a citation and full reference for the retraction notice.

**E1-1: We thank the editor for the positive evaluation of our manuscript and for the opportunity to resubmit our manuscript.**

Reviewer 1:

I think the manuscript is in a very good shape in its current form and the authors have done an excellent job to address the points I raised in my previous review. I particularly want to praise the thorough robustness analyses that have been conducted in response to my earlier comments and the introduction of a new example that much better demonstrates the application of the methodology. I do have a few really minor comments on the current version, particularly on the new example application, but in my view, these should not impede the publication of the manuscript.

**R1-1: We thank the reviewer for the very positive evaluation of our revised manuscript.**

In the application example, the manuscript reads “It can be seen that all Bayes factor trajectories cross the upper threshold of 30 at some point”. However, Figure 4 seems to suggest that the trajectories cross the threshold of 20, but not of 30? Is there potentially an issue with the axis labels? Furthermore, it may be helpful to very briefly discuss the definition of stopping thresholds in the new paragraph about the sequential design (i.e., why is 30 a good choice?)

**R1-2: We are grateful that the reviewer caught this error. It turned out that the figure is correct but the text is wrong. That is, we used a threshold of 20. We have corrected the text accordingly. Further, we followed the reviewer's request to give some additional explanation about stopping thresholds. We wrote the following: “In general, the choice for a specific decision threshold should be tailored to the research at hand. There are scenarios where more certainty is desired compared to other scenarios. However, researchers should be aware of the tradeoff between certainty and resource demands; the more certain we want to be, the more cases must be sampled.”. We think that a more detailed discussion about this extensive topic is beyond the scope of our article.**

In the example application, is there any benefit of using the data-driven priors compared to the default priors? Judging based on Figure 4, it looks a bit like the data-driven priors yield Bayes factors in the upper third of the distribution, which would indicate at least some efficiency benefit over the default priors. I understand that the authors are reluctant to point to the potential efficiency benefits of informed priors (I’ve gathered as much from the response to the second reviewer). However, I think the application example shows that at least IF the parameter in the present study is similar to the corpus of literature used for deriving the prior, then efficiency gains can be expected compared to SOME default priors. Moreover, the variability in data-driven priors is much smaller than in the other priors, potentially indicating greater stability of results if priors are chosen in a principled manner.

**R1-3: In general, we are very much in favour of using informed priors when relevant information is available. In our case, however, we did not observe efficiency gains of the data-driven priors. Although based on eyeballing, it appears that the data-driven priors result in Bayes factors in the upper third of the distribution generated by the default priors used in the sensitivity analyses, numerical confirmation revealed that it is closer to the median. In an additional analysis, we looked at potential Bayes factor decision thresholds of {3, 4, …, 60}. For almost all of these Bayes factor decision thresholds, the rank of the data-driven priors, in terms of the speed of reaching that threshold, was in the middle, compared to the default priors we used in our sensitivity analysis. In other words, roughly 50% of the priors reached the decision thresholds faster and 50% slower. We observed that in our scenario, priors that are more concentrated at 0 (i.e., smaller standard deviation) reached the decision thresholds faster.**

In Figure 4, it is difficult to distinguish the red and blue lines. Is there any way to overcome this issue (perhaps decreasing the line width and/or making the grey lines in the background brighter would help?), or if not, could the authors perhaps briefly note the overlap in the figure caption so that readers don’t get confused?

**R1-4: To mitigate this issue, we have now decreased the line width and increased the transparency of lines. These steps improved the visibility of the lines. However, since the blue and red lines overlap so heavily, the visual distinction is still not perfect. Therefore, we added another note to the caption of Figure 4: “The blue and red lines heavily overlap, so the blue line is hardly visible.”**

In response to my earlier comment R1-7, the authors added a section to the introduction that contains the sentence “A prominent way to deal with this issue within the frequentist framework is the use of sequential designs” – I think this should be group sequential designs?

**R1-5: We have now corrected this sentence according to the reviewer's request.**

I thought the new figure illustrating the article retention rate across processing steps was interesting – will this be included in the appendix / supplementary materials?

**R1-6: We have now included and explained the figure in an appendix.**
